# Reduction of visual stimulus artifacts using a spherical tank for small, aquatic animals

**DOI:** 10.1038/s41598-021-81904-2

**Published:** 2021-02-05

**Authors:** Kun Wang, Burkhard Arrenberg, Julian Hinz, Aristides B. Arrenberg

**Affiliations:** 1 Werner Reichardt Centre for Integrative Neuroscience, Institute for Neurobiology, University of Tübingen, 72076 Tübingen, Germany; 2 Graduate Training Centre for Neuroscience, University of Tübingen, 72076 Tübingen, Germany; 3 Prudenter Agas Hamburg, 22149 Hamburg, Germany; 4 Present Address: Friedrich Miescher Institute for Biomedical Research, 4058 Basel, Switzerland

**Keywords:** Visual system, Neuroscience, Optics and photonics, Experimental organisms, Imaging, Microscopy, Ichthyology

## Abstract

Delivering appropriate stimuli remains a challenge in vision research, particularly for aquatic animals such as zebrafish. Due to the shape of the water tank and the associated optical paths of light rays, the stimulus can be subject to unwanted refraction or reflection artifacts, which may spoil the experiment and result in wrong conclusions. Here, we employ computer graphics simulations and calcium imaging in the zebrafish optic tectum to show, how a spherical glass container optically outperforms many previously used water containers, including Petri dish lids. We demonstrate that aquatic vision experiments suffering from total internal reflection artifacts at the water surface or at the flat container bottom may result in the erroneous detection of visual neurons with bipartite receptive fields and in the apparent absence of neurons selective for vertical motion. Our results and demonstrations will help aquatic vision neuroscientists on optimizing their stimulation setups.

## Introduction

In vision research, an important standard protocol is to present visual stimuli to immobilized animals while recording behaviors and/or neuronal activity^1–6^. The visual system extracts features from stimulus patterns, and, accordingly, visual neurons can respond to a range of features such as contrast, motion direction, spatial frequency, sizes, locations and shapes of the visual stimulus^5,7–11^. Therefore, presenting high quality visual stimulus patterns to the animal eyes is crucial for the investigation of visual functions and neural encoding^12^.

A conventional mounting platform, equipped with a standard-size monitor or a small LCD screen, is suitable for neural direction selectivity and receptive field (RF) mapping analysis of the vertebrate optic tectum^7,13^, since the RFs of most tectal neurons are small^13–15^ and visual stimulus parameters, such as contrast and spatial frequency, are easy to control with programmable hardware and software^16,17^.

Due to the large RFs of some of the neurons as well as the large binocular fields of view in the lateral-eyed zebrafish^18,19^, the stimulus delivery system needs to cover a large proportion of the visual space surrounding both eyes. Ideally the stimulus surface should completely cover their visual fields, and the platform supporting the animal should allow for a large accessible view field for the animals^20–22^.

Furthermore, research using aquatic model animals (e.g. fish) gives rise to additional challenges, since an underwater environment is required during the experiments^20,23^. Electronics need to be kept outside of the water, and therefore visual stimuli are oftentimes blurred and distorted before they reach the animal eyes, which mainly results from light refraction and reflection and the associated geometrical optics at the air-container-water interfaces^12,24^. For example, during the presentation of global optic flow, the motion consistency across the visual field may be disrupted. Alternatively, the stimulus delivery system could be set underwater, surrounding the experimental animal. However, the waterproof protection for the electronic setup is usually difficult to ascertain and it may cause optical disturbances itself.

Here, we make use of Snell’s law, which posits that disruptive optical effects are quite small when light beams pass through an optical interface orthogonally, and design a new spherical glass bulb container, coupled with an adjustable rotation mount holder to optimize vision experiments in small-size fish. Moreover, we demonstrate the optical advantages of the new glass bulb using optical simulations and functional neural activity recordings.

## Results

### Design and optical advantages of a spherical glass container

**Figure 1:**
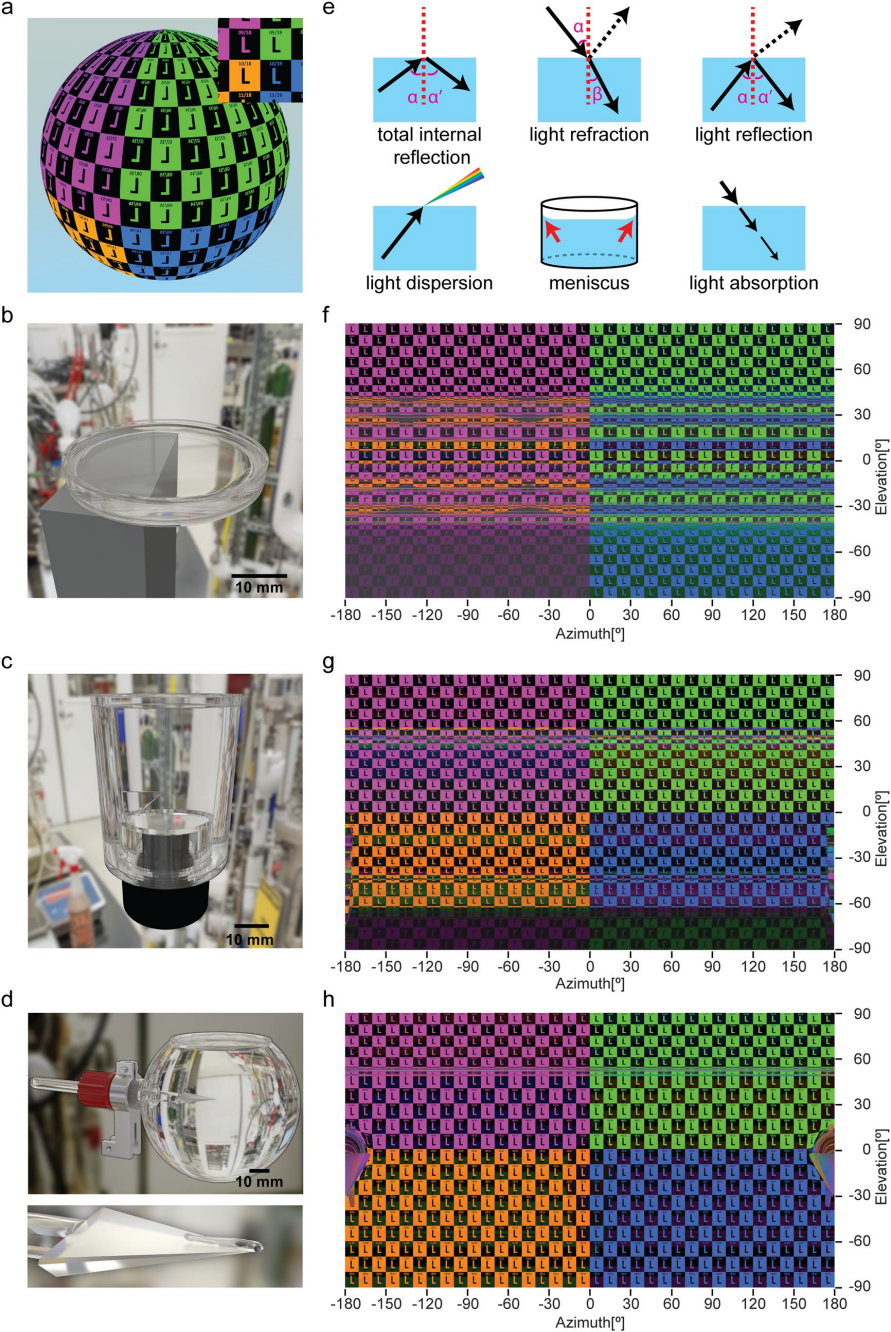
Simulation of visible stimulus patterns in three different water containers. (**a**) The sphere shows the simulated checkerboard stimulus pattern (18 rows, 36 columns) under the ideal optical condition. The letter “L” was used to keep track of the orientation (up, down, left, and right) of stimulus patches. Inset: a patch of the checkerboard stimulus (0° in elevation; 0° in azimuth) seen from the inner center of the container. (**b–d**) Photorealistic illustrations of three water containers (3D rendering). (**b**) A commercially available Petri dish lid (Ø 38.7 mm) on a metal stage. (**c**) A plastic cylindrical container made by a fine mechanics workshop (Ø 40 mm) on a camera lens holder. (**d**) A custom-made glass bulb (Ø 80 mm) attached to a holder. Below, a simulated larval zebrafish is on the tip of a triangular stage. (**e**) Illustration of optical effects: total internal reflection, light refraction, reflection, dispersion, water meniscus, and light absorption. Black arrows represent light beams and vertical red dashed lines indicate the perpendiculars. Angles α, α’, and β indicate angles of incidence, reflection and refraction. Red arrows indicate the water meniscus at the container wall. (**f–h**) 360° panorama picture of the simulated visual patterns seen from the perspective of the animal in the center of the corresponding containers. (**f**) Petri dish lid, (**g**) cylindrical container, and (**h**) glass bulb.

The underwater presentation of visual stimuli can suffer from a range of optical artifacts, such as total internal reflection (TIR), light refraction, general reflection, dispersion, water meniscus, and light absorption (Fig. 1e). Given the different refractive indices of water, air, and the container material, the shape of the container (e.g. flat vs. round walls) will have a profound impact on the path of the light transitioning into water. In many previous studies on zebrafish vision, the potential occurrence of stimulus artifacts had received little attention^12^, although superior container designs had already successfully been used for other fish species^25^. Here, we compare the occurring optical artifacts of three different containers for aquatic animals: a commonly used Petri dish lid (Fig. 1b), a cylindrical water container (Fig. 1c), and a new spherical glass container that we designed to minimize artifacts (Fig. 1d). The glass bulb container is 8 cm in diameter and has an opening of 4.8 cm (in diameter) on the top, which allows for in vivo microscopy using a water immersion objective from above (Fig. 1d, Supplementary Figure S1). During the experiment, the animal is immobilized on the tip of a triangular stage located in the center of the spherical glass bulb (Supplementary Figure S1). In comparison to the Petri dish lid and the cylindrical container (Fig. 1b,c and Supplementary Figure S1), the spherical glass bulb allows for larger homogeneously accessible visual space (see the spherical panorama views covering 360° in azimuth and 180° in elevation in Fig. 1f–h). In addition, using a rotation mount metal holder (Supplementary Figure S1), the animal’s position is adjustable around the three axes of the Cartesian space.

### Simulation of optical stimulus artifacts in different containers

We simulated the details of the visual stimulus patterns for the three water containers as perceived by the fish's eyes (Fig. 1f–h, Table 1). In the simulation, the upper visual space was covered by two quarter spheres of purple and green colored checkerboard stimuli. The lower visual space was covered by orange and blue stimuli (Fig. 1a).

**Table 1:** Dimensions and observer positions for the three containers.

	Petri dish lid	Cylindrical container	Glass bulb
Container height (diameter)	4 mm	50 mm	72 mm
Water level in the container	About 2.3 mm, close to the rim	50 mm, filled up	72 mm, filled up
Height of the animal’s eyes in each container	Less than 1 mm above the bottom of the Petri dish lid	About 1.75 mm (above the triangular stage)	About 1.75 mm (above the triangular stage)
Camera position in the simulation	0.5 mm (above the bottom of the Petri dish lid)	1.75 mm (above the triangular stage)	1.75 mm (above the triangular stage)
Height of the animal relative to the LED arena	The center of the LED arena; at the half height level of the LED arena		

Strong artifacts are present for Petri dish lids around the equator (− 41.4° to + 41.4° in elevation, Fig. 1f). For example, in our simulation the animal’s right eye sees a pattern of alternating green and blue stimuli, so stimuli from the upper and lower visual space are inappropriately mixed. These stimulus artifacts result from total internal reflection (TIR) and are visible to animals placed inside the Petri dish lid (Fig. 1f). Any light ray that penetrates into the water through the air-plastic-water interface of Petri dish side wall with an incidence angle smaller than 62° (Supplementary Figure S2) is subject to TIR. Furthermore, the visual space above the water surface (and at the flat Petri dish bottom) is compressed into visual field elevations ranging between roughly 41.4° and 90° (and roughly − 41.4° to − 90° for the Petri dish bottom), which corresponds to the so-called Snell’s window. Thus, in very flat water containers such as the Petri dish lid, visible stimuli in the elevation range around the equator (roughly − 40° to + 40°) correspond to stimulus light rays that entered the water body through the side walls of the Petri dish. Many of these light rays are subject to multiple rounds of TIR reflection at the water surface and the Petri dish bottom before they reach the animal’s eye (Supplementary Figure S2). These TIR stimulus artifacts are absent in the spherical container (Fig. 1h) and in our cylindrical container (Fig. 1g).

Furthermore, the stimulus pattern observed by the animal’s eye in the Petri dish lid gets distorted via refraction when the light beams intersect the air-to-water border, i.e. at the water surface, or through the bottom of the Petri dish lid. Strong refraction is observed for large incidence angles (α) of light (Fig. 1e), which causes stimulus compression along the vertical axis at elevations around 50° (Fig. 1f), while stimuli near the poles (close to + / − 90° elevation) still have the squarish aspect ratio present in the undisturbed checkerboard stimulus. These refractive distortions are also present in the cylindrical container (Fig. 1g, elevation ranges from approx. 30° to 70° and − 30° to − 70°). In the spherical container, the opening for the microscope objective still causes refractive distortions for elevation angles exceeding 60° (Fig. 1h). However, for the spherical container this upper part of the visual field would in any case be blocked by the microscope objective during experiments requiring microscopy (Supplementary Figure S1, elevation angles exceeding 39.2° are blocked).

In addition to TIR and refraction, general light reflection artifacts occur in all three containers on both the contralateral and ipsilateral sides of the presented visual stimuli. Due to the similar refractive indices of the containers (Table 2), the reflectance on the contralateral side is quite similar for all three containers. The reflectance is relatively low, for small incidence angles of light (Petri dish lid, 5%; cylindrical container, 4%; glass bulb, 4%). Though weak, the contralateral reflection is still visible to the contralateral eye on a dark background (Supplementary Figure S4). The effects of the reflection can influence monocular experiments as shown experimentally further below.

**Table 2:** The materials and the corresponding indices of refraction of the three containers.

Containers	Petri dish lid	Cylindrical container	Glass bulb
Material	Polystyrene	Acrylic glass	Glass Schott Boro
IOR	1.57	1.49	1.473

Reflection on the ipsilateral side is very weak in case of the glass bulb and the cylindrical arena (0.14% and 0.16, respectively). In the Petri dish lid, however, the ipsilateral reflectance is high (first reflection approx. 25%, second reflection, approx. 15%) when light rays come from above or below. For example, in the lower visual field (− 40° to − 90° in elevation) of the left eye (0° to − 180° in azimuth), a reflection of the upper visual space (purple-colored stimuli) is faintly visible (Fig. 1f). Furthermore, depending on the water level in the Petri dish lid, these lateral reflections can be partially blocked by the objective due to its small access angle. In contrast, the reflection resulting from the light rays directly entering from the side is negligible for the Petri dish lid as well (0.29%).

Light dispersion is restricted to non-orthogonal light beams passing through the water surface (Fig. 1e and Supplementary Figure S1). Therefore, it does not occur in the glass bulb since only orthogonal light beams reach the visual field of the stimulated animal eye via the bulb. For the Petri dish and cylindrical container, light disperses on the non-spherical polystyrene surfaces. Blur and rainbow edges resulting from chromatic aberration are visible to the animal (Supplementary Figure S1). The light dispersion artifacts are, however, very weak in comparison to reflection and refraction artifacts, especially when working with narrow-bandwidth (monochromatic) LEDs (Supplementary Figure S1).

Modelling the surface of the water close to the edge of the containers (Fig. 1e and Supplementary Figure S1) revealed that the water meniscus only interrupts the narrow range of upper elevations (e.g. 50° in elevation in the spherical container, Fig. 1h and Supplementary Figure S1). Due to the relatively shallow access angles of microscope objectives (e.g. 39.2°, Supplementary Figure S1), the water meniscus and rim do not affect the upper visual field of the fish in the spherical and cylindrical containers. For the Petri dish lid, the meniscus artifacts are visible to the animal, but they are much less problematic than the above described light refraction and reflection artifacts (Fig. 1f).

Next to directional changes of light rays, which were discussed above, a fraction of the light also gets absorbed in the water. Due to the small sizes of the containers, light absorption within pure water does not exceed 0.2% for green stimulus light^26^ and, therefore, is negligible for our analysis. While the transparency of the three containers compared here is similar, edges or thickenings of the container (e.g., at the vent edges and the outer reinforcement ring of the Petri dish lid, or at the glued faces of the cylindrical tank increase opacity Fig. 1b,c and Supplementary Figure S1).

The zebrafish has a large monocular visual field of about 160° and the accessible visual space is even larger when taking eye movements into account^27,28^. For each container in this study, its holder blocked parts of the stimulus (Figs. 1f–h). Accordingly, a compact design of the holder is recommended to minimize the visible holder silhouette. For example, in our Petri dish lid simulation, the lid holder blocks the stimulus presented in the lower visual space of the left eye. Instead, reflections of the stimulus in the upper part are visible in the animal’s lower left visual field region (magenta instead of orange colors, Fig. 1b,f). Due to the camera and bracket mounted at the bottom of the cylindrical container, the visual field between − 45° and − 60° is disrupted, and downwards from − 60° (in elevation) it is blocked for all azimuth angles. Similarly, stimulus reflections of the upper view field are visible to the animal in this blocked visual field region (Fig. 1c,g). In contrast, for the spherical container, the mount holder, the glass rod (stage holder), and the wedge-shaped glass stage only occlude a small region of visual space in the rear of the animal (Fig. 1d,h and Supplementary Figure S1).

In summary, the new glass bulb designed in this study offers a better optical environment to present visual stimuli to small aquatic animals than the other two containers. There is no disruption by TIR or light refraction and remaining reflection is very weak. Furthermore, stimuli covering a larger range of visual space can be presented using the glass bulb.

### Total internal reflection (TIR) disturbs receptive field mapping experiments in the optic tectum

**Figure 2:**
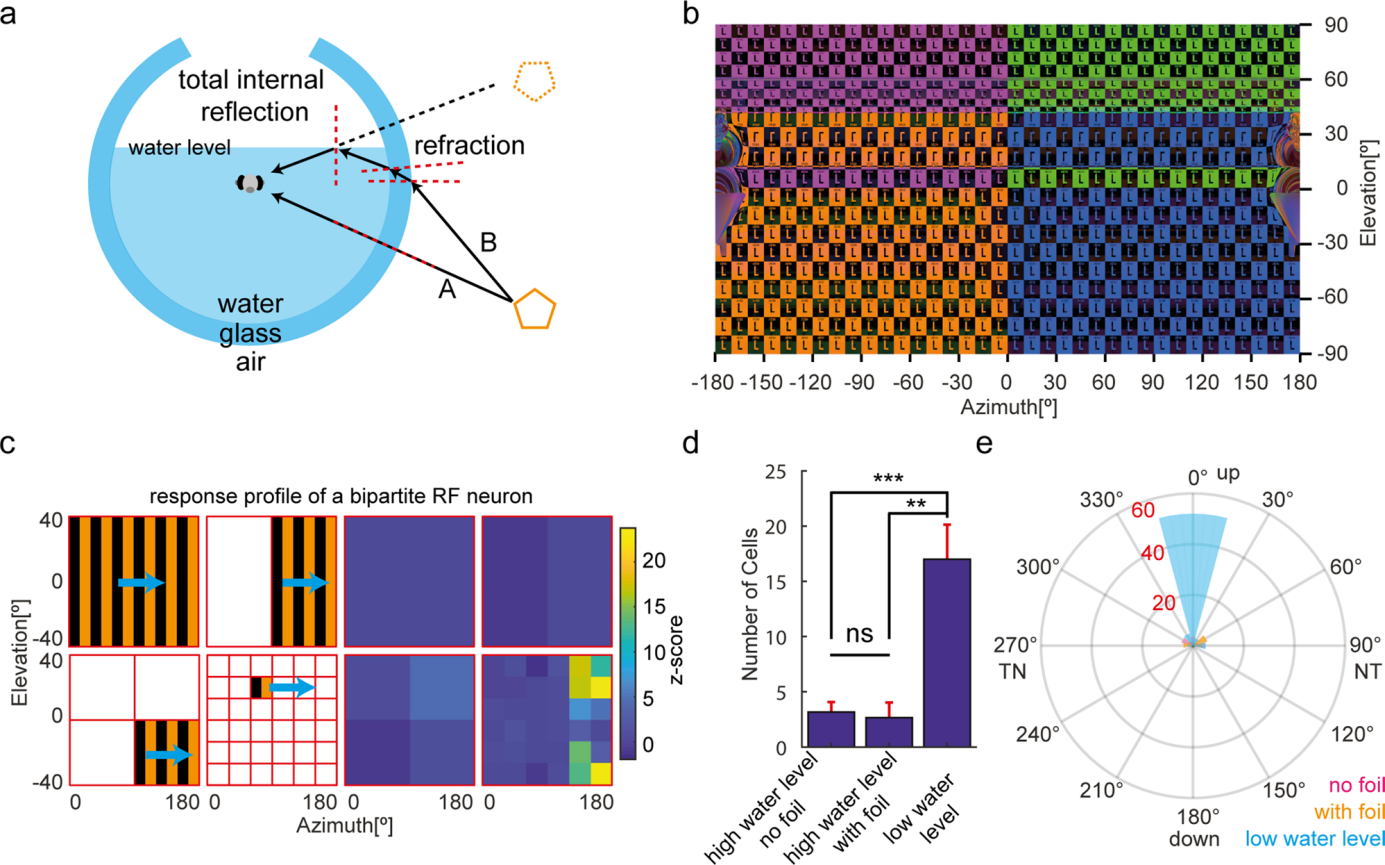
TIR results in the erroneous detection of bipartite receptive fields in the zebrafish optic tectum. (**a**) Illustration of TIR for the glass bulb with low water levels. Light beam A is perpendicular to the air-glass-water interface and reaches the fish right eye directly. However, light beam B, from the same light source as light beam A, is refracted twice on the air-glass-water interfaces and then reaches the fish's eye from above via TIR. Black arrows, light beams; dashed red lines, perpendiculars; orange pentagon, one object; dashed orange pentagon, reflection of the object. (**b**) A 360° panorama picture of the stimulus pattern seen from the glass bulb center with low water level. Part of the visual stimulus below the equator is reflected to the upper visual field (the upside-down letters "L" in orange and blue) via TIR. (**c**) The response profile of a bipartite RF neuron. Left side: a diagram of the monocular RF mapping protocol (Supplementary Figure S3 shows the whole protocol). Horizontally moving gratings (indicated by cyan arrows) of different sizes and locations were sequentially presented to the right eye of the animal. Right side: z-scores of the calcium signal of a bipartite RF neuron responding to motion phases on the left. The neuron responded exclusively to small-size motion. Two separate RF centers are present in the same neurons and located in the upper and lower temporal visual fields, respectively. (**d**) The average numbers of bipartite RF neurons in each tested fish measured under different experimental conditions. ***, *p* < 0.001; **, *p* < 0.01; ns, no significant differences. The “high water level” group was further split into two subgroups (cf. Figure 3) for which the left eye was either occluded (“with foil”) or left free to see potential contralateral reflections (“no foil”) (**e**) A polar histogram of the orientation of the two RF centers for each neuron. Orientations of 0° and 180° correspond to a vertical orientation, 90° and 270° to a horizontal orientation of the bipartite RF centers. TN, temporal-nasal direction; NT, nasal-temporal direction. Neuron numbers are indicated in red. n = 6 (3 composite fish brains, high water level no foil), 6 (3 composite fish brains, high water level with foil) and 4 (2 composite fish brain, low water level) recordings in (**d**) and (**e**).

When the water level is low, TIR occurs even in the spherical container (Fig. 2a) or in the cylindrical container (Supplementary Figure S3) and thus influences the visual perception of the fish. Stimuli presented at certain positions below the equator are reflected and visible to the animal in its upper visual field (Fig. 2a), resulting in stimulus inconsistencies above the equator (Fig. 2b). To demonstrate the effects of TIR on vision experiments, we mapped the receptive fields (RFs) of zebrafish tectal neurons using calcium imaging with a two-photon microscope^8^.

In the first condition, we filled a glass bulb (Ø 10 cm) up to 11.5° in elevation (1 cm water level above the animal, Fig. 2a). In the second condition, we filled a glass bulb (Ø 8 cm) completely with water (up to ~ 53° in elevation; Table 1). We mapped visual RFs of larval zebrafish tectal neurons by using horizontally moving gratings of different sizes and locations that were presented to the right eye of the animal (Fig. 2c and Supplementary Figure S3, see “Methods”). While most motion-sensitive tectal RFs should have a single, unimodal RF according to previous work^8^, in the low water level condition (with visible TIR) some of the neurons (68 out of 543) appeared to have bipartite (double-field) RFs with two RF centers (Fig. 2c,d). The two RF centers were vertically aligned for the vast majority of these bipartite RFs (Fig. 2e). Therefore, this “bipartite RF” type is most likely a TIR artifact resulting from light reflection at the horizontal water surface (Fig. 2a,b).

Tectal small-size RF centers (defined as cells with excitatory RF areas smaller than 1170 deg^2^, also see Data analysis), cover nearly the whole monocular visual field in larval zebrafish and are biased to the upper nasal visual field (Supplementary Figure S3)^8^. In this hotspot region, however, only very few small-size tectal RFs were identified when the glass bulb was not filled up with water completely (Supplementary Figure S3). The small number of detected neurons was likely a combined result of TIR, reflection and water meniscus stimulus artifacts.

### General reflection disturbs monocular receptive field mapping experiments

**Figure 3:**
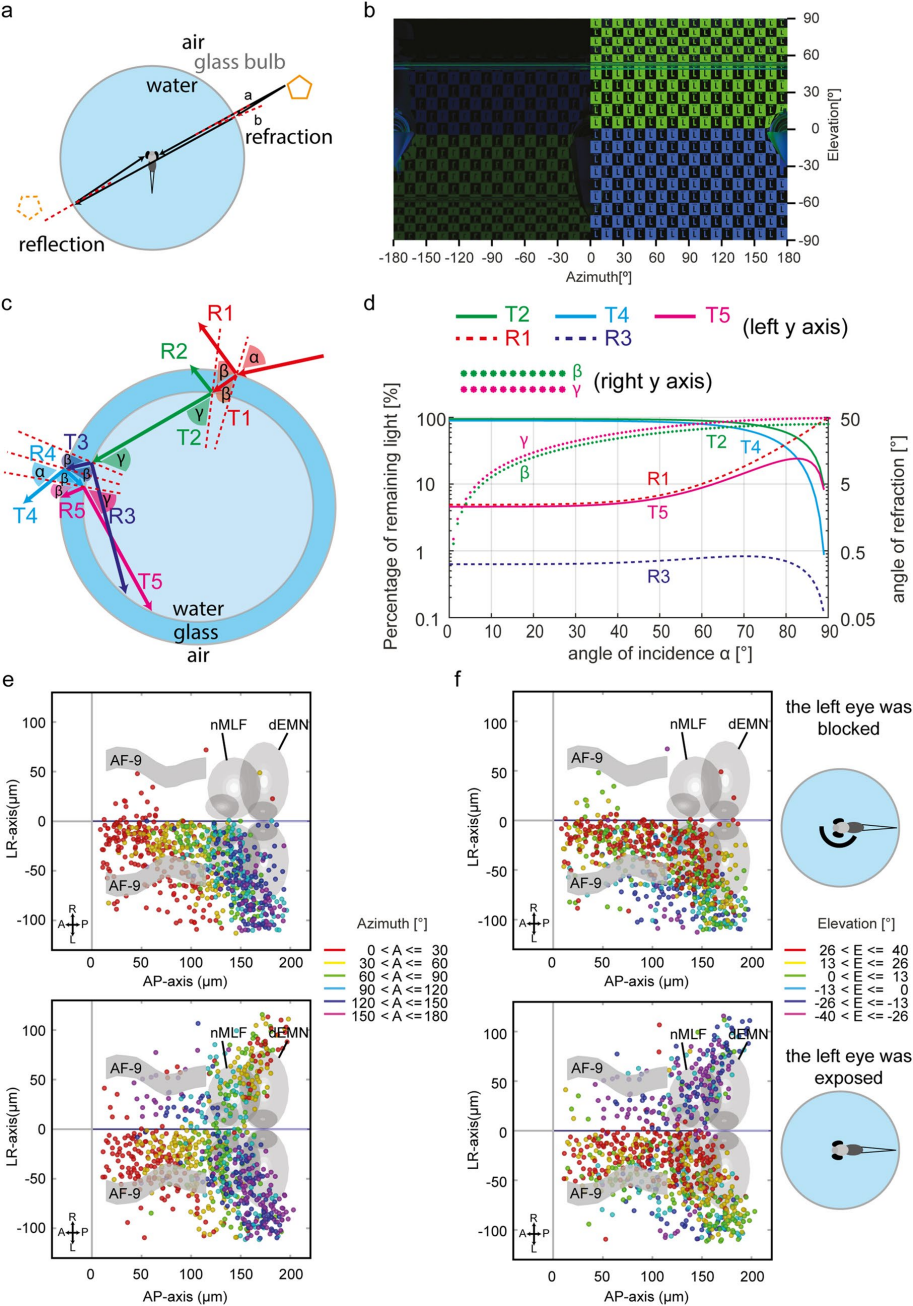
Remaining reflections in the glass bulb are visible to the animal. (**a**) Beam a, perpendicular to the air-glass-water interface, reaches the fish’s right eye directly and is absorbed. Light beam b, with a small but non-zero incidence angle at the air-glass-water interface, passes by the fish and is reflected back to the “non-stimulated” left eye. (**b**) 360° panorama picture of a half sphere stimulus: In the left hemifield (0° to − 180° azimuth), the “non-stimulated” eye can see the point-symmetric reflection (left) of the monocular stimulus (right). (**c**) Detailed illustration of light reflection and refraction in the glass bulb. Angles α and β, angles of incidence and refraction on the air-glass interface; γ, the refraction angle at the glass-water interface. Since the glass is thin and the angle β is relatively small, the incidence angle at the glass-water interface approximates refractive angle β at the air-glass interface. Only light beams with relatively strong high power are shown here. Light beams R3 and T5 can reach the “non-stimulated” eye when the incidence angle α is small. ‘R’ and ‘T’, reflected and transmitted light beams. (**d**) Final reflection and refraction rates of the light, and angles of refraction calculated according to the Fresnel equation and Snell's law. Some of the light beams in (**c**) have roughly equal light intensities and were therefore omitted in the plot in (**d**): T1 = T2 = T3; R2 = R3 = R5; R4 = T5. (**e–f**) Topographic maps [in azimuth (**e**) and elevation (**f**)] of RF centers of small-size RF tectal neurons in the zebrafish (dorsal views). The “non-stimulated” (left) eye was covered in the first row (control group) but exposed to potential stimulus artifacts below (experimental animals). Each colored dot represents a single neuron with its receptive field center in the corresponding azimuth or elevation range. For example, neurons with RF centers between 0° (in front of the fish) and 30° azimuth on the nasal right side of the fish are in red in (**e**). n = 6 fish for both groups, 3 composite brains.

As discussed above, reflections of the stimulus are visible for the animal on the contralateral side (Fig. 3a,b). Any “visible” point of the stimulus display emits light rays that reach the ipsilateral eye (light beam a in Fig. 3a), as well as light rays that miss the ipsilateral eye, part of which can be seen as reflection by the contralateral eye in the spherical container (light beam b in Fig. 3a). The visible reflected light intensity is only about 4% of the original light (Fig. 3c,d, Fresnel equations), since the visible light rays hit the air-glass-water interface nearly perpendicularly (e.g. light beam A in Supplementary Figure S5). The Petri dish and cylindrical containers suffer from similar reflections (Supplementary Figure S4).

We tested the influence of the visible reflection to the “non-stimulated” eye using tectal RF mapping and a spherical container completely filled with water. In the control group, the non-stimulated eye (left eye) was blocked by a black foil. In these control animals, the active tectal neurons were mainly located in the tectal hemisphere contralateral to the stimulated eye as expected from the complete midline crossing of retinal ganglion cell axons in the optic chiasm. The RF centers furthermore showed the expected topographical distribution along the anterior–posterior, medial–lateral, and dorso-ventral axes (Fig. 3e, f and Supplementary Figure S4). In contrast, in the experimental group without occlusion of the non-stimulated eye, many tectal neurons were identified in the ipsilateral hemisphere as well, and these ipsilateral neurons distributed in the tectum according to a reverse topographic map (Fig. 3e,f and Supplementary Figure S4). These results suggest that the left eye (the “non-stimulated” eye) saw the weak stimulus reflection and that this reflection was strong enough to activate a small proportion of neurons in the corresponding tectal hemisphere. The roughly point-symmetric nature of this reflection also explains the occurrence of a topographic map with apparent reverse order (Fig. 3e,f and Supplementary Figure S4).

### TIR and light refraction in the Petri dish lid result in inaccurate detection of preferred directions in zebrafish tectal neurons

**Figure 4:**
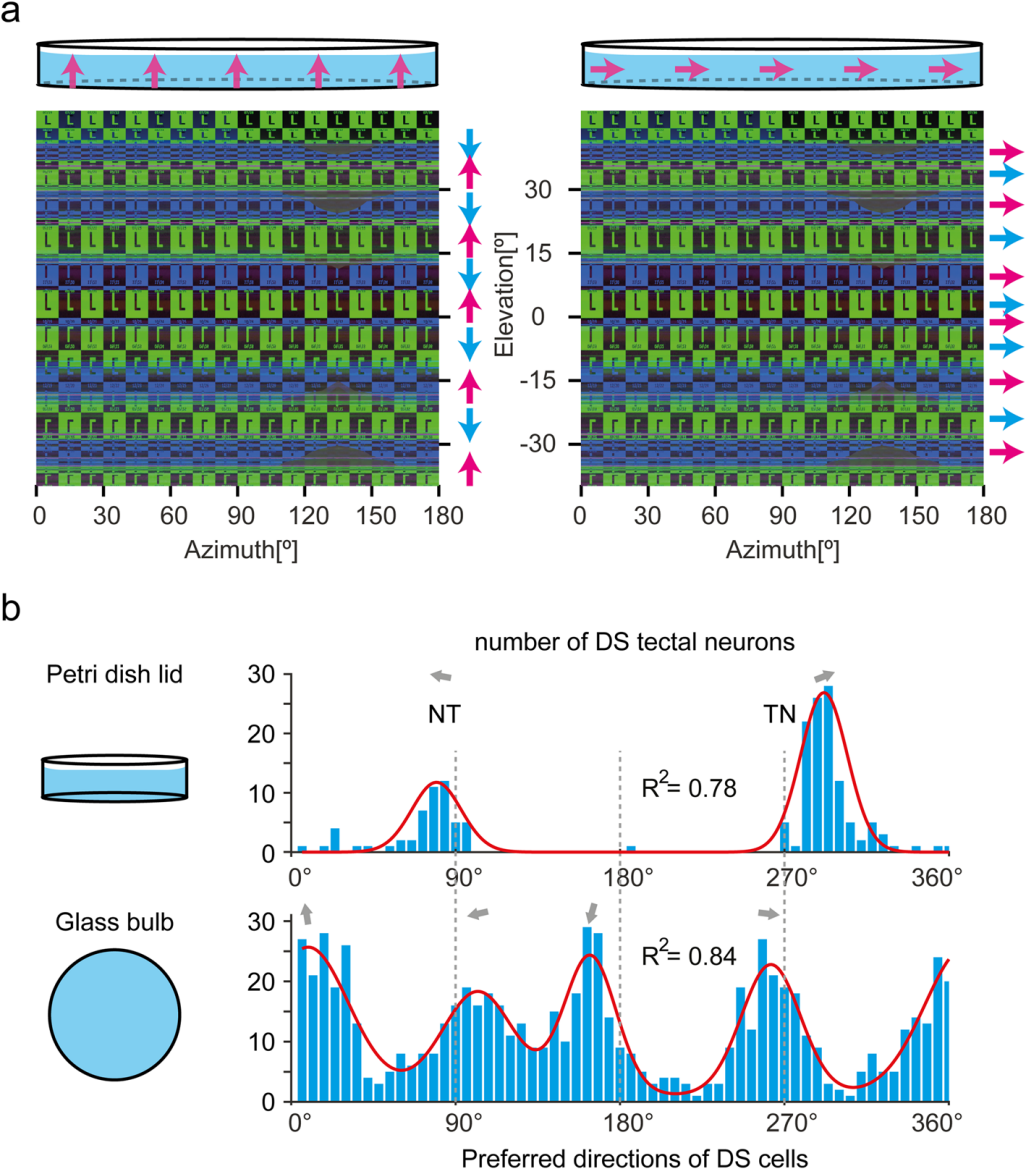
Vertical motion stimuli are disrupted due to TIR. (**a**) 180° panorama of the right side of the stimulus seen from the center of a Petri dish lid. Due to TIR, the visual stimulus patterns near the equator (− 41° to 41° in elevation) are intermingled and oftentimes oriented upside-down (background colors and letters ‘L’, also see Fig. 1f). Left side: vertical upward motion (indicated by magenta arrows) is presented from outside of a Petri dish. The whole moving pattern is disrupted and not continuous from the perspective of the animal in the center of the container. At different elevation levels of the visual field, the stimulus patches move upwards (magenta arrows) or in the opposite direction (cyan arrows). Right side: horizontal motion (indicated with magenta arrows) to the right is presented from outside of a Petri dish lid. Seen from the center of the Petri dish, the whole moving pattern is still continuous, consistent and homogeneous (cyan and magenta arrows). (**b**) Histograms of the preferred directions of direction-selective tectal neurons recorded in a Petri dish lid (top, n = 5 fish) or a glass bulb (bottom, n = 9 fish). The peaks were fitted with a sum of two (top) or four (bottom) Von-Mises functions (red lines). The PDs are indicated with gray arrows.

The inconsistency and disruption of the visual stimulus patterns in the Petri dish lid are mainly caused by TIR at the horizontal interfaces. In vision experiments, this should lead to stimulus artifacts of different severity for vertical and horizontal motion directions. For shallow elevation angles in the Petri dish, each light ray bounces within the water before reaching the fish's eye and the bounce number depends on the vertical angle of view. For even and odd numbers of reflection, the vertical moving directions of the visual stimuli projected to the fish's eye are opposite. As a result, the vertical motion of simple grating bars is seen as motion in opposite vertical directions for odd numbers of TIRs (Fig. 4a). Clearly, this should strongly reduce responsiveness of vertical direction-selective neurons that have a large receptive field. In contrast, such direction-inverting stimulus artifacts should be completely absent for horizontal motion directions of the stimulus, since these motion directions are parallel to the TIR-inducing water surface and lid bottom (Fig. 4a).

To demonstrate how direction selectivity analysis gets compromised by this artifact, we presented motion in eight different directions using a Petri dish lid (in this study) or a glass bulb as the container for the animal (Supplementary Figure S3)^29^. Using the glass bulb, we appropriately detected direction-selective tectal neurons, which preferred either vertical or horizontal directions. For each of the four preferred directions (PD: up, down, nasalwards, and temporalwards), we found an approximately equal number of neurons in the tectum^29^. In contrast, but as expected from our optical analysis above (Fig. 4a), almost no direction-selective neurons preferring upward or downward stimulus motion could be detected in the experiments using the Petri dish lid. The histogram of preferred directions was instead dominated only by two peaks, nasalward and temporalward directions. The two PD peaks point a little upward compared to the corresponding horizontal PDs recorded with the glass bulb. The temporal-nasal direction (292°, 109 cells) is represented by many more neurons than the opposite nasal-temporal direction (80°, 44 cells; z-test for proportions, *p* < 0.00001) (Fig. 4b).

## Discussion

Our study identifies a number of optical stimulus aberrations caused by suboptimal water levels, tank geometry or illumination in commonly used containers for aquatic animals in vision research. These stimulus aberrations can have alarmingly adverse effects on the outcomes of RF mapping and direction selectivity analysis, as we show in calcium imaging experiments. Therefore, careful experimental design is needed for aquatic vision experiments. We provide a solution in the form of a spherical glass container that can minimize most of the identified optical artifacts.

### The glass bulb offers a better optical presentation of the visual stimulus than the other two containers

Visual stimuli seen from within the Petri dish lid are severely disrupted across about 80° in elevation near the equator by TIR (Fig. 1f). The bouncing light beams (Supplementary Figure S2) cause three caveats. First, visual stimuli from different spatial locations lead to stimulus overlap and blurring when they are projected to the same region within the fish's eye. Second, stimuli from adjacent spatial locations project to angular regions far away in the eye (e.g., 30°), disrupting the stimulus pattern from the perspective of the animal. Third, at several elevation levels of the visual field, stimuli are mirrored by reflections (Fig. 1f). For a vertical whole-field motion stimulus, this results in visible opposing vertical directions (Fig. 4a) and a corresponding loss of direction-selective neuronal responses. Furthermore, light refraction leads to vertical stimulus pattern compression for certain elevation levels. Therefore, the Petri dish lid is a suboptimal container for aquatic animal vision research, especially for experiments including presentation of vertical motion or small-size stimuli in specific locations used for RF mapping or prey capture experiments.

The cylindrical container offers a better optical environment than the Petri dish lid, though a large part of the visual field is disrupted by light refraction and reflection (Fig. 1g). A cylindrical container should be designed to be tall enough (high on the top and deep at the bottom) to prevent TIR artifacts and minimize the disturbance by its holder, water meniscus, and media interfaces. Note that refraction artifacts can largely be corrected digitally by pre-adjusting the stimulus within the presentation software. Furthermore, refraction artifacts can be reduced by rear-projecting the stimulus onto the side wall of the water container. In experiments where stimuli only need to be presented in small parts of the visual field, e.g. for certain studies on zebrafish prey capture, the combination of cylindrical containers with digitally pre-warped stimulus patterns can therefore enable high-quality stimulation^30^. Using a spherical glass container, however, dramatically minimizes distortion and blur of the visual stimulus across the entire visual field of the animal (Fig. 1h). While the spherical glass container outperforms cylindrical containers and Petri dish lids, several potential caveats still persist. First, it is important to place the animal in the center of the glass sphere, since mispositioning the fish in the horizontal plane away from the spherical center results in stimulus distortion near the two poles (Supplementary Figure S5). Second, the custom manufacture of glass bulbs with a perfect spherical curvature and homogeneous thickness can be difficult. Fortunately, the visual stimulus quality is quite robust against inaccurate manufacturing (Supplementary Figure S5). Third, our monocular RF mapping experiment revealed that the low reflectance (4% of the original intensity) at the glass-air interface is strong enough to activate tectal neurons. Occlusion of the non-stimulated eye is strongly recommended in monocular experiments with the glass bulb or with any water container. A further reduction of reflectance could potentially be achieved by optical coatings. Alternatively, the inner surface of the glass could be coated with a diffusive paint and used as a rear projection screen for the stimuli. The diffusive effects of the rear projection screen (water container in this case) can dramatically reduce the effects of light reflection, and therefore would improve the stimulus quality of the glass bulb further.

Our glass bulb is suitable for small aquatic animals. For larger fish, the diameter of the glass bulb could be increased. Previous receptive field mapping experiments in the fish tectum mainly depended on placing electrophysiological equipment close to the animal^25,31,32^, which oftentimes limited the available visual space for unobstructed presentation of stimuli. When using a water-dipping objective and calcium imaging, a visual space cone of roughly 100° in diameter remains blocked by the objective. Our receptive field mapping results showed a similar topographic map as recorded in other fish species before^25,31,32^. Since a half-cylindrical arena was used in our experiments, we were unable to measure the entire extent of the visual field of zebrafish larvae, since our arena only subtended 80° in the vertical direction. In future experiments a full-surround visual stimulus display^33^ could be used to take full advantage of the glass bulb water container design.

### Influence of geometrical optics on RF mapping and direction selectivity analyses

Many apparently bipartite RF neurons were detected in the larval zebrafish tectum when the water level in the glass bulb was low and allowed TIR to be visible (Fig. 2). However, not all of the small-size RF tectal neurons with RF centers above the equator responded to the reflection (Supplementary Figure S3). We speculate that the low intensity and contrast of the reflection—relative to those of the cardinal stimulus—prevented these neurons from being detected as bipartite neurons in our analysis.

In our investigation of direction-selective neurons, only two out of the four previously reported neuronal populations of direction-selective neurons were detectable when a Petri dish lid was used for the experiment (Fig. 4b)^29^. The incomplete detection most likely resulted from the disruption of the vertical motion by TIR (Fig. 4a).

In summary, we demonstrate that optical artifacts can disturb key parameters such as motion directionality, and they can displace the position of the visual stimulus in aquatic vision experiments. Use of a spherical water container can greatly reduce artifacts. Our results showcase potential pitfalls in experimental design and provide a roadmap for careful design of vision experiments in aquatic environments.

## Methods

### 3D visualization techniques

All 3D visualizations were performed with the 3D computer graphics software Blender v2.79b and v2.9 (https://www.blender.org). The different experimental environments were modelled in detail and rendered in pictures to illustrate different effects. In this study, geometrical optical effects resulting from light transmission (absorption), reflection (including TIR), refraction, dispersion and occlusion, were calculated, simulated, and visualized.

#### Containers

We simulated three different fish containers based on popular usage in zebrafish vision research with dimensions corresponding to those of the real objects (see Supplementary Figures S6 and S7).

- Petri dish lids (Greiner 627,102, Fig. 1b)
  The panoramic camera was positioned 0.5 mm above the bottom of the lid, to simulate the zebrafish that was embedded using agarose, at the bottom of the lid^34^.
- Cylindrical container (Fig. 1c)
  The panoramic camera was positioned 1.75 mm above the point of the stage, to simulate the zebrafish that was immobilized on a small, custom-made stage in the middle of the cylinder, by low-melting agarose.
- Glass bulb (Fig. 1d)
  The panoramic camera was positioned 1.75 mm above the point of the stage, to simulate the zebrafish that was immobilized on a small triangular stage, which was then placed in the center of the water-filled glass bulb^8^.

The different mounting devices were modeled together with their respective containers and a water level just below the rim of the container (see Table 1).

#### Inputs

The main inputs, indices of refraction (IOR) for different containers, are listed in Table 2 for the photorealistic modeling.

#### Stimulus

For optical simulations, a colored checkerboard pattern, consisted of 36 columns and 18 rows, was used with different colors to differentiate the main directions (up/down and right/left, marked by a letter ‘L’ to identify mirroring).

#### Renderer and cameras

The images were usually rendered using the Cycles renderer of Blender. For photorealistic representation we mostly used an f = 35 mm perspective camera (e.g., Fig. 1b–d). For the test of the optical quality by pictures of the stimulus out of the fish's point of view (with 360° azimuth × 180° elevation) we used a panoramic equirectangular camera (e.g. Figure 1f–h) with output in UHD format (3840 × 2160 px). The symbolic representations for illustrations were made as screen snapshots (e.g., Supplementary Figure S1).

Only in special tasks, such as the visualization of the light beam path and the analysis of dispersion, images were rendered with an orthographic camera in LuxCoreRender v2.1 (https://luxcorerender.org/download/) using bidirectional path tracing and appropriate volume scattering and camera clipping (e.g., Supplementary Figure S1, S2)^35,36^.

## Physical background

**Table:** 

Snell's Law	${\mathrm{n}}_{\mathrm{i}}\mathrm{sin}{\upvarepsilon }_{\mathrm{i}}={\mathrm{n}}_{\mathrm{t}}\mathrm{sin}{\upvarepsilon }_{\mathrm{t}}$	The angle of incidence (ε_i_) and the angle of refraction/transmission (ε_t_) with given indices of refraction n_i_ and n_t_ for two media
Fresnel equations	${\mathrm{R}}_{\mathrm{s}}= {\left|\frac{{\mathrm{n}}_{\mathrm{i}}\mathrm{cos}{\upvarepsilon }_{\mathrm{i}}-{\mathrm{n}}_{\mathrm{t}}\mathrm{cos}{\upvarepsilon }_{\mathrm{t}}}{{\mathrm{n}}_{\mathrm{i}}\mathrm{cos}{\upvarepsilon }_{\mathrm{i}}+{\mathrm{n}}_{\mathrm{t}}\mathrm{cos}{\upvarepsilon }_{\mathrm{t}}}\right|}^{2}$	R_s_, the reflectance for s-polarized light
Fresnel equations	${\mathrm{R}}_{\mathrm{p}}= {\left|\frac{{\mathrm{n}}_{\mathrm{i}}\mathrm{cos}{\upvarepsilon }_{\mathrm{t}}-{\mathrm{n}}_{\mathrm{t}}\mathrm{cos}{\upvarepsilon }_{\mathrm{i}}}{{\mathrm{n}}_{\mathrm{i}}\mathrm{cos}{\upvarepsilon }_{\mathrm{t}}+{\mathrm{n}}_{\mathrm{t}}\mathrm{cos}{\upvarepsilon }_{\mathrm{i}}}\right|}^{2}$	R_p_, the reflectance for p-polarized light
	${\mathrm{R}}_{\mathrm{eff}}=\frac{1}{2}({\mathrm{R}}_{\mathrm{s}}+{\mathrm{R}}_{\mathrm{p}})$	R_eff_, the total reflectance of unpolarized light
	$\mathrm{T}=1-\mathrm{R}$	T, the fraction of the transmitted power; R, the fraction of the reflected power;

### Limits of the 3D modeling technique

Because of the limitation of our 3D modeling approach, some physical properties of the stimulation setup have not been tested in our simulations. These properties include light polarization, light interference, and diffraction. Refraction and reflection can change the polarization of light, and the intensity of the reflected light furthermore depends on the polarization of the incident light. Light interference should be absent in our experimental setup or only cause stimulus artifacts at very small spatial scales. Diffraction does not occur for the container shapes in our setup, which is in the macroscopic range well above the wavelength of visible light. Therefore, light interference and diffraction effects are not relevant for the experiments in question here.

### Animal care and transgenic lines

Animal experiments and all experimental protocols were approved by the responsible ethics committee of the Regierungspräsidium Tübingen in accordance with German federal law and Baden-Württemberg state law. The study was carried out in compliance with the ARRIVE guidelines.

The transgenic zebrafish lines *Tg(HuC:GCaMP5G)a4598Tg* was used in this study*.* Transgenic lines were kept in either a TL or TLN (nacre) background. Zebrafish larvae were raised in E3 medium until day 5 or 6 post-fertilization (dpf).

### Experiment protocols and data analysis

We performed the monocular direction selectivity and monocular RF mapping experiment as described previously^8,29^, except that we used a Petri dish lid (depth of the lid, 4 mm; water level in the lid, about 2.3 mm) as a container instead of the glass bulb in the direction selectivity experiments. The data shown in the bottom of the Fig. 4b have been published before as the bottom part of the Fig. 1e in^29^.

#### Immobilization of animals and calcium imaging

On the day of experiments, a triangular stage (Supplementary Figure S6), cleaned with pure water and dried, was coated with poly-lysine and dried. Then the triangular stage was inserted into a ‘T’-shaped glass holder to keep the triangular stage in position during mounting. The firm connection between the triangular stage and the holder was accomplished using conically tapered ground glass joints, with the inner cone (male) and the outer socket (female) attached to the triangular stage and the holder, respectively. The connected stage and glass holder were then transferred into an agarose mold (2% agarose) prepared in a large-size Petri dish (Supplementary Figure S6). A small plastic ring (outer diameter, 20 mm; inner diameter 16 mm; height 3 mm; printed with a 3D printer) was positioned onto the triangular stage, with the center of the ring overlapping with the triangular tip, to make a small well for the animal (Supplementary Figure S6). The cavity surrounding the triangular stage created by the agarose mold and the ring was filled with low-melting agarose (1.6%). Before the liquid low-melting agarose in the cavity cooled down and began to form a solid gel, a larval zebrafish (5 or 6 dpf) was transferred onto the triangular stage in a drop of low-melting agarose using a Pasteur pipette. Using a thin platinum wire tool^37^, the position of the larva was adjusted, so that the eyes protruded both sides of the triangular tip to allow free view of the surrounding visual space. The position of the larva could be adjusted by pushing or pulling the agarose near the animal using the wire (i.e. touching the animal as little as possible). E3 water was added to the large-size Petri dish after the agarose gel had formed, in order to keep the animal alive. After a waiting period of 5 min, the ring was gently removed and the low-melting agarose around the triangular stage was cut to free the stage with the animal mounted on it. Then the ‘T’-shaped holder with the triangular stage was lifted from the mold with one up-forward motion. The low-melting agarose around the animal can be further truncated in E3 water to minimize the refractive and absorptive effects of the agarose if necessary. Then the triangular stage (with the fish) was pulled out of the socket of the holder, transferred into the empty glass bulb and inserted into the socket of the glass rod holder. The glass bulb was filled up with E3 water and fixed onto the metal holder with an M4 screw (Supplementary Figure S7).

The position of the larva was adjusted to the center of the half-cylindrical LED arena (ideally 92 mm away from the arena (184 mm in diameter) and 240 mm above from the experiment platform (post length: 160 mm, half-height of the arena: 80 mm)) via the metal holder and the glass rod holder (Supplementary Figure S7). The setup allowed independent adjustment of roll and rostro-caudal position via the shaft of the glass bulb and/or the glass rod holder, translation-invariant pitch adjustment via the arced, sliding mount fixed by the golden screw (Supplementary Figure S7e–f). Translation-invariant yaw corrections could be made using the arced long slot of the assembly post at the base of the breadboard (Supplementary Figure S1c). Then the objective (magnification, 20x, numerical aperture: 1.0, Zeiss, 421,452-9880-000) was attached to the objective holder and adjusted to illuminate the animal using the blue excitation light of the microscope’s widefield GFP channel to generate visible green GCaMP5G fluorescence. We first adjusted the pitch, yaw and roll position of the fish coarsely and then more precisely using 2-photon imaging directly before the recording started (Supplementary Figure S7).

The stimulus patterns were visually checked and confirmed before they were shown to the animals. The microscope, recording glass bulb and stimulus arena were enclosed in a dark box in a dark room. In case of the light reflection from the surface of metal in the dark box, the opening side of the cylindrical LED arena was covered by a large black foil. Furthermore, in monocular stimulus experiments, we covered the non-stimulated eye of the animal with a half-cylindrical black foil.

In this study, glass bulbs with diameters of 8 cm or 10 cm were used. In case of the 10 cm diameter bulb, the water level was only 1 cm above the glass bulb center.

#### The half-cylindrical LED arena for visual stimulation

Visual stimuli were presented to zebrafish using a half-cylindrical LED arena assembled from 7168 LEDs (Kingbright TA08-81CGKWA): 8 (rows) × 14 (columns) × 64 (8 × 8 multiplexed LED matrix) LEDs. Since the metal holder of the glass bulb stage protruded into the space of the most caudal column, the LEDs in the caudal-most column of the half arena were removed (i.e. 14, not 15 columns). Correspondingly, the view field covered by the half arena ranged from 0° to + 168° (-168° to + 168° for the whole arena) in azimuth and − 40° to 40° in elevation. The LEDs’ spectrum peaked at 570 nm and an additional high-pass filter foil (LEE no. 779, article 595-1700-7790, castinfo.de, Hagen, Germany) and diffusion filter foil (LEE no. 252, article 595-1780-2520) filtered and diffused light to optimize GCaMP signal detection and to homogenize the emitting light. Measured from the position of the animal without fish containers, the luminance values of the visual stimulus presented with the LED arena are 28.0 ± 0.73 and 2.7 ± 0.42 cd/m^2^ in the bright and dark areas of the gratings, respectively.

The half-cylindrical LED arena, with an inner diameter of 184 mm, height 160 mm, was fixed on three 160 mm high posts onto the experimental platform.

To provide hardware control to the LEDs, we used circuit boards designs and C controller software code provided by Alexander Borst (MPI of Neurobiology, Martinsried) and Väinö Haikala and Dierk Reiff (University of Freiburg)^38^. The electronic and software architecture of stimulus control has originally been designed by Reiser et al.^17^, the documentation is available at https://bitbucket.org/mreiser/panels/wiki/Home.

#### Calcium imaging

The calcium signals from the larval tectum were recorded with a two-photon MOM microscope (Sutter Instruments, Novato, California) and a Coherent Vision-S Ti-Sa laser (set to 920 nm) while presenting the visual stimulus three times in pseudo-random orders with the half-cylindrical LED arena, which was controlled using custom-written Matlab (R2015b) scripts. Between each recording, the imaging was stopped for a pause lasting 2–3 min. During the recording of the time series, the frame rate, image size, and the pre-pulse compensation of the laser were set to 2 frames per second, 512 × 512 pixels and 9756 fs^2^.

We used two visual stimulus protocols in this study, one for the direction selectivity analysis and the other one for receptive field mapping (Supplementary Figure S3). In the former protocol, whole field moving gratings in eight different directions were presented to the right eye of the animal. In the latter protocol, horizontally moving gratings (0.033 cycles/°, moving at 30°/s in temporal-nasal direction or reversely) with different sizes (the smallest size being 30° azimuth × 13° elevation, corresponding to 1/36 of the whole view field covered by the half cylindrical LED arena) and locations were shown to the animals. See Wang et al.^8^ for a more detailed description of the stimulus protocol and analysis. The posterior commissure was used as the landmark (z = 0 µm). We recorded the calcium activities from the imaging layers + 60 µm (below the landmark) to − 80 µm (above the landmark) with increments of 10 µm (i.e. no recording at e.g. 5 or 15 µm below the landmark). Since the second visual stimulus protocol was very long, we sampled the optical imaging layers with an increment of 20 µm. Then we merge the data from two fish recorded from complementary layers (i.e. one fish was recorded from layers + 10 µm, + 30 µm etc. and the other from 0 µm, + 20 µm etc.) as one combined brain. An anatomical z-stack of the recorded brain region was recorded with an increment of 0.43 µm for the 3D registration of the recorded neuron at the end of the experiment.

### Data analysis

Data analysis was performed with published Matlab scripts (MOM_Load, R2014b; Midbrain_Localizer and Cell_Viewer, R2010b), available online (https://gin.g-node.org/Arrenberg_Lab)^8,29,34,39^. Briefly, the ROIs were manually selected in recorded optical sections using a heat map in which the stimulus-correlated image pixels were colored.

In the direction selectivity analysis, we calculated the orientation and direction selectivity indices (OSI and DSI) of all identified motion-sensitive neurons. All neurons with a DSI higher than 0.7 were classified as direction selective neurons.

In the receptive field mapping experiment, our smallest moving stimulus covered 30° × 13° (azimuth x elevation) of the visual field. Limitations on recording time and the focus on comparing tectal RFs to the very large RFs in the pretectum precluded us from including stimuli smaller than 1170 deg^2^. We classified the RF sizes broadly into 5 subclasses in our previous publication^8^. Here, for our investigation of stimulus artifacts, only the small-size receptive field neurons in the tectal region were of interest and are plotted in the figure. The smallest stimulation field (30° × 13° in azimuth and elevation) was used as a calculation unit (i.e. 1 “patch”). 3 patches (1170 deg^2^) were arbitrarily set as the threshold. Therefore, a small-size receptive field covered 3 or fewer patches of our smallest stimulation fields.

The 3D registration of the recorded neurons from 2D optical imaging layers to the 3D z-stack recording of each brain was performed with the Midbrain_Localizer and Cell_Viewer Matlab (R2010b) algorithms to visualize the distribution of these neurons.

### Quantification and statistical analysis

The statistical information (calculated with Matlab R2014b built-in functions) is provided in each of the sections above. For statements of significance an alpha level of 0.05 (two-tailed) was used unless stated otherwise.

The analyzed number of zebrafish and brains is indicated in the main text and Figure legends. Error bars correspond to SEM unless stated otherwise.

## Supplementary Information

Supplementary Information

## Data Availability

All raw and processed data and custom-written Matlab (R2010b, R2014b and R2015b, https://www.mathworks.com/products/matlab.html) software used to generate the Figures will be made available upon request.
