# Supplementary material for: Reduction of visual stimulus artifacts using a spherical tank for small, aquatic animals
Source: Sci Rep. 2021 Feb 5;11:3204. doi: 10.1038/s41598-021-81904-2 (PMC7864920; doi:10.1038/s41598-021-81904-2)
Supplement: Supplementary file 1 — Supplementary Information [file 41598_2021_81904_MOESM1_ESM.pdf]

Supplementary Figures

Title:

Reduction of visual stimulus artifacts using a spherical tank for small, aquatic animals

Authors:

Kun Wang[1,2,§], Burkhard Arrenberg[3,§], Julian Hinz[1,2,4], and Aristides B Arrenberg[1*]

Affiliation

[1] Werner Reichardt Centre for Integrative Neuroscience, Institute for Neurobiology, University of Tübingen, D-72076 Tübingen, Germany

[2] Graduate Training Centre for Neuroscience, University of Tübingen, D-72076 Tübingen, Germany

[3] Prudenter Agas Hamburg, 22149 Hamburg, Germany

[4] Current address: Friedrich Miescher Institute for Biomedical Research, 4058 Basel, Switzerland

[§] these authors contributed equally

Correspondence

* Correspondence should be addressed to A.B.A (aristides.arrenberg@uni-tuebingen.de)

Figure S1. Three different containers for fish vision research and simulation of geometrical optics. Related to Figure 1

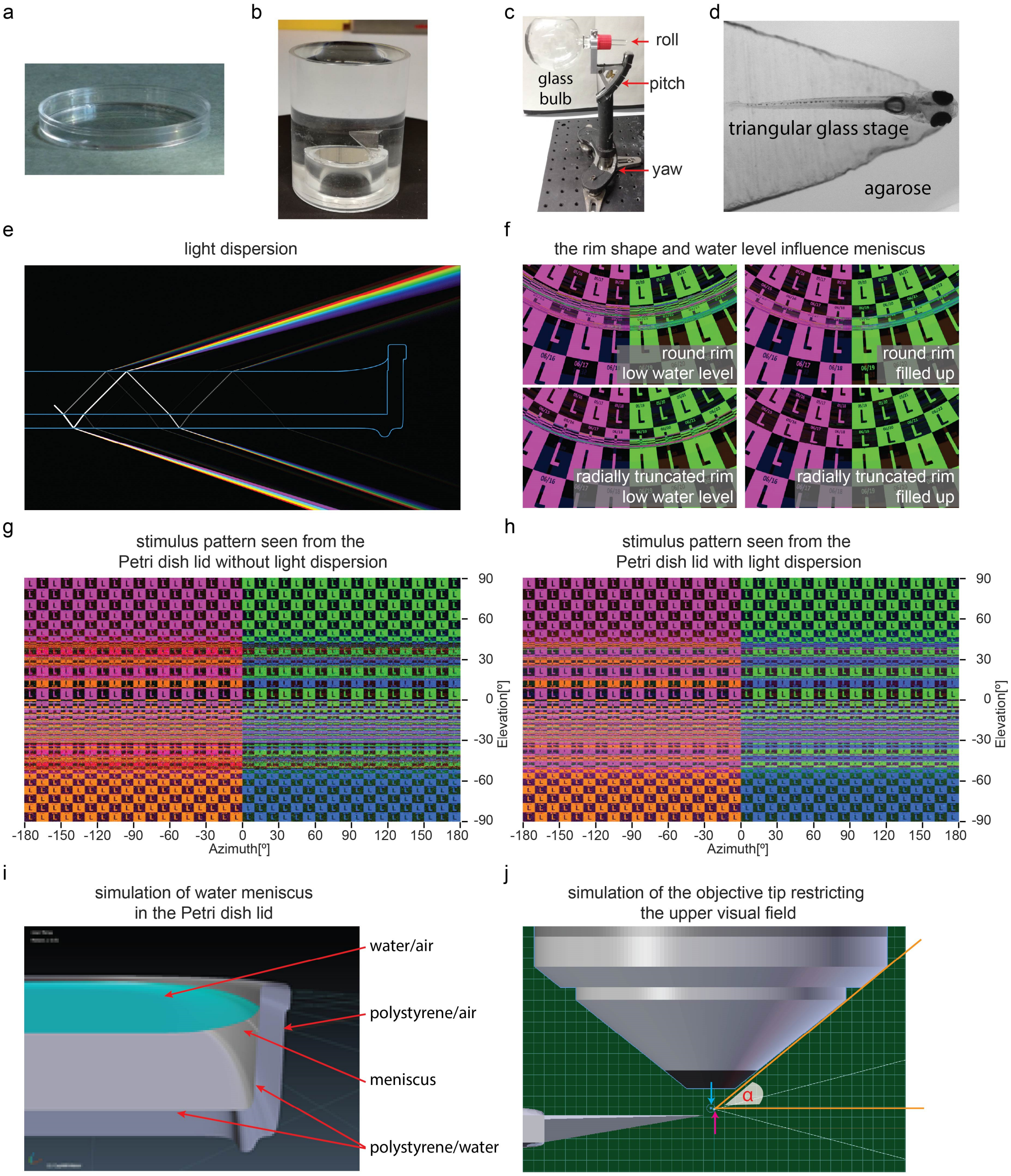

Figure S1. Three different containers for fish vision research and simulation of geometrical optics. Related to Figure 1

(**a**) A commercially available Petri dish lid (Ø 38.7 mm). (**b**) A plastic cylindrical container made by a fine mechanics workshop (Ø 40 mm). (**c**) A custom-made glass bulb (Ø 80 mm) attached to its metal holder which allows for rotational adjustment (roll, pitch, and yaw) around three axes. (**d**) A 5 dpf larval zebrafish is embedded in low-melting agarose on a triangular stage in the glass bulb. (**e**) A white light beam is shed 45° downwards in the center of a Petri dish lid (side view). Because of different indices of refraction from the white light components, light dispersion occurs at the water-air and plastic-air interfaces. (**f**) The rim shape and water level influence the water meniscus in the glass bulb. Using radial truncated rim and filling up the glass bulb with water reduce the optical influences of water meniscus. The viewing perspective is from the center of the glass bulb to the stimulus point at 0° in azimuth and 50° in elevation. (**g**) The checkerboard stimulus seen from the center of the Petri dish lid (without the holder stage) without light dispersion. (**h**) The checkerboard stimulus seen from the center of the Petri dish lid (without the holder stage) with light dispersion. Color distortions and blurring exist in comparison to panel (g) but their influences are weaker than those of light infraction and reflection. (**i**) A symbolic simulation of water meniscus in the Petri dish lid (side view). On the inner wall of the Petri dish lid, the water level is higher than more central regions. (**j**) The accessible angle α (orange angle = 39.2°) of the fish to the visual stimulus is only about 1° larger than the angle of the objective tip (38.4°) since the location of the fish's eyes (magenta arrow) are roughly 0.3 mm in front of the focus point (cyan arrow) of the objective (Zeiss, 421452-9880-000, numerical aperture, 1.0; magnification, 20x; free working distance, 1.7 mm; diameter, 35 mm).

Figure S2. Simulation of the optics underlying visual stimulus distortion and blurring resulting from light refraction and reflection in the Petri dish lid. Related to Figure 1

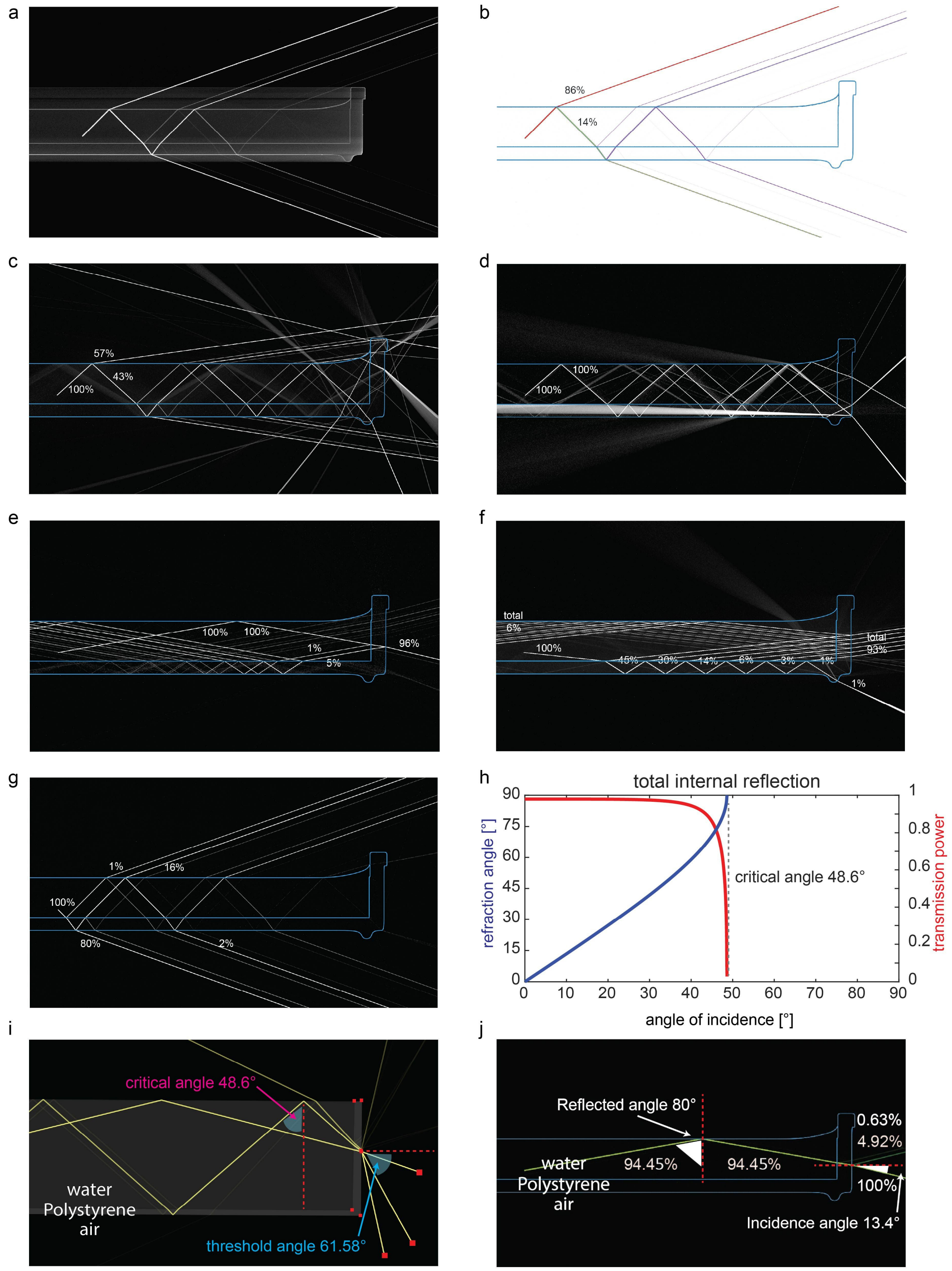

Figure S2. Simulation of the optics underlying visual stimulus distortion and blurring resulting from light refraction and reflection in the Petri dish lid. Related to Figure 1

For each panel from (a) to (g), only one single light beam is shown under different conditions (angles of incidence and directions). The beam splits in several rays. Because of the reversibility of the light path, this shows what the fish will see when looking in the same direction as the beam. The split rays indicate directions from which the fish will see stimulus parts. The several parts will mix to the picture for the fish with the given power of light. (**a**) A light beam is shed 45° (applicable from 0° to 48.6°) upwards. 86% of the light is refracted into the air and only 14% is reflected back. Light reflections and refractions continue to the Petri dish side wall. (**b**) The same as in panel (A) except that the light intensity is color coded (red to purple representing 100% to 2% of original light power). (**c**) A light beam is shed 48° (applicable from 0° to 48.6°) upwards. 57% of the light is refracted into the air and already 43% is reflected back. Light reflections and refractions continue to the Petri dish side wall. (**d**) A light beam is shed 49° (larger than critical angles, 48.6° from water to air and 39.6° from polystyrene to air) upwards. The light beams cannot be refracted from water or polystyrene to air. (**e**) The same as in panel (d) except that the light beam is shed 80° upwards. The light beam can only come out of the Petri dish lid through the vertical lid wall. (**f**) A light beam is shed 84° downwards. (**g**) A light beam is shed 45° downwards. (**h**) Refraction angle (in blue) and transmission power (in red) change when a light beam is projected from water (IOR = 1.333) to air (IOR = 1) and the angle of incidence increases from 0° to 90°. The critical angle of total internal reflection from water to air is indicated with a vertical dashed line. (**i**) TIR occurs on the water-air interface of the Petri dish lid when a light ray comes from below with an incidence angle smaller than 61.58°. The reflected light can reach the fish's eye depending on the point of incidence and incidence angle. (**j**) An example light ray from panel (i). When a light ray enters the Petri dish lid with an incidence angle of 13.4° from below, 4.92% and 0.63% total light energy will be reflected on the outer and inner wall of the lid, while a light ray with 94.45% incidence light energy is totally reflected on the water-air interface.

Figure S3. Experimental protocols for monocular direction selectivity analysis and RF mapping; low water level disrupts the distribution of receptive field centers for small-size RF tectal neurons measured with the glass bulb. Related to Figures 2, 3 and 4

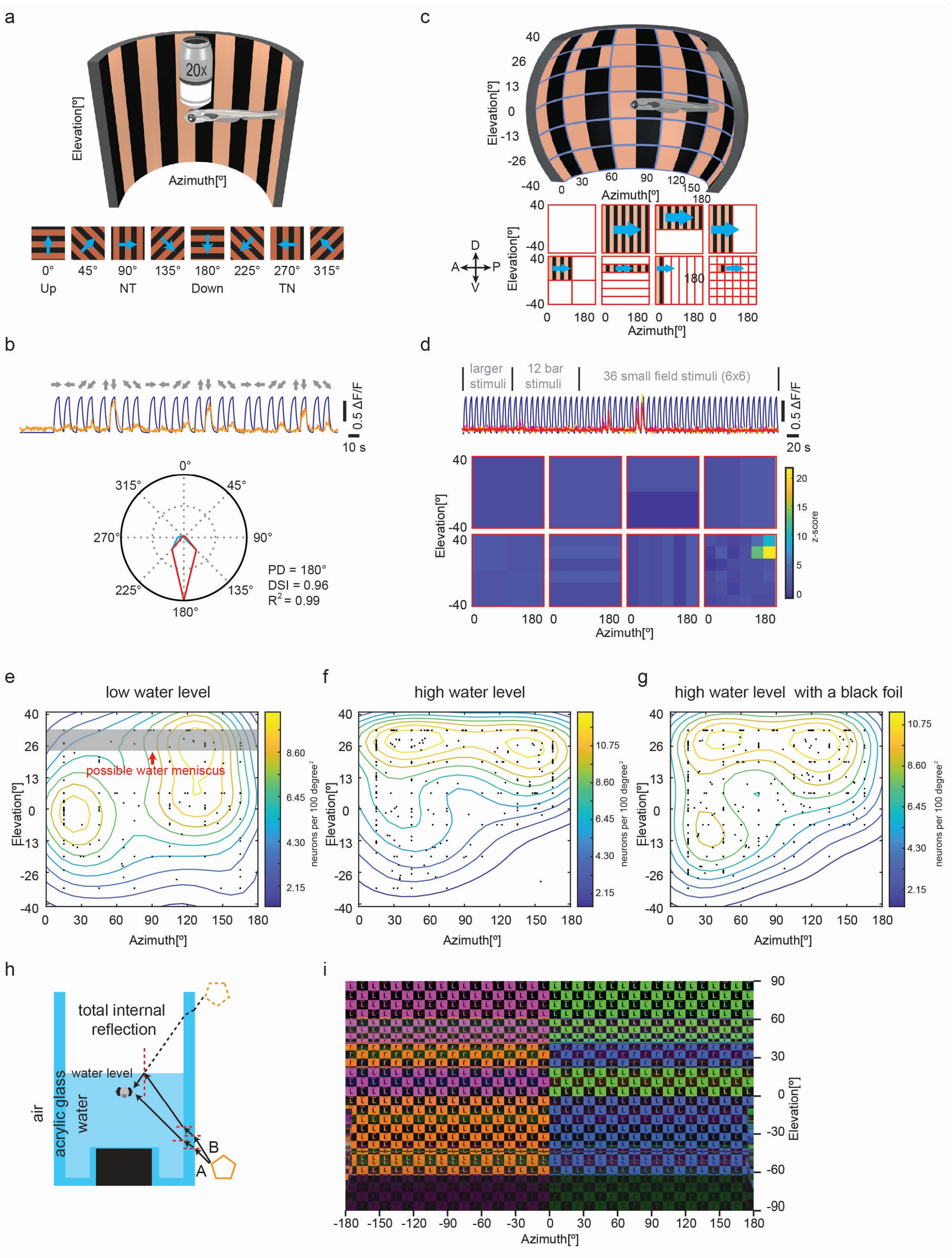

Figure S3. Experimental protocols for monocular direction selectivity analysis and RF mapping; low water level disrupts the distribution of receptive field centers for small-size RF tectal neurons measured with the glass bulb. Related to Figures 2, 3 and 4

(**a**) A diagram of the monocular direction selectivity analysis setup. Upper, gratings, 0.033 cycles per degree, moving in eight different directions, were presented to the right eye of the animal, which was embedded in low-melting agarose in the center of the cylindrical half arena. The sizes of the fish and the arena are not proportional to the real experiments in this illustration. Below, an illustration of the 8 moving patterns. The cyan arrows indicate the moving directions. NT, nasal-temporal; TN, temporal-nasal. (**b**) An example direction-selective (DS) tectal neuron. Upper, the original calcium trace of the DS neuron in three repetitions (in orange). The blue background curve indicates the convolved (τ = 1.5 s) motion-stationary phase regressor. Below, the DS tuning curve. The neuron responded most robustly to the downward motion. (**c**) A diagram of the monocular RF mapping protocol. Upper, vertical gratings (0.033 cycles per degree) with different sizes and locations were presented to the right eye of the animal, which was embedded in low-melting agarose in the center of the cylindrical half arena. The sizes of the fish and the arena are not proportional to the real experiments in this illustration. Below, an illustration of the whole visual stimulus protocol. The cyan arrows indicate the moving directions. A, anterior; P, posterior; D, dorsal; V, ventral. (**d**) The response profile of a small-size RF neuron plotted z-score. Upper, the original calcium traces (yellow, cyan and magenta) of the neuron in the three repetitions and their median (orange). The blue background curve indicates the convolved (τ = 1.5 s) motion-stationary phase regressor. Below, the response profile of a small-size RF neuron plotted with z-score. The response in each phase is corresponding to one of the motion phases in panel (c). (**e-g**) Visual field locations and density contour plot of receptive field centers of small-size RF tectal neurons recorded with low water level (panel (e), n = 4 fish, 2 composite tecta), high water level without left eye covered (panel (f), n = 6 fish, 3 composite tecta) and high water level with left eye blocked by a black foil (panel (g), n = 6 fish, 3 composite tecta), respectively. In panel (e), the low neuron density in the upper visual field potentially resulted from water meniscus (gray shade). (**h**) An illustration of TIR for the cylindrical container with a low water level. Light beam A reaches the fish right eye directly after refraction on the interfaces of air-glass and glass-water. However, light beam B, from the same light source as light beam A, is refracted twice like the beam A and then reaches the fish's right eye from above via TIR. Black arrows, light beams; dashed red lines, perpendiculars; orange pentagon, one object; dashed orange pentagon, reflection of the object. (**i**) A 360° panorama picture of the stimulus pattern seen from the cylindrical container center with low water level. Part of the visual stimulus below the equator is reflected to the upper visual field (the upside-down letters "L" in orange and blue)

via TIR. The view in the darker region below is blocked by the container holder and in that region the animal sees the reflection of the upper part of the visual stimulus.

Figure S4. Reflections in the glass bulb activated tectal neurons corresponding to the non-stimulated eye. Related to Figure 3

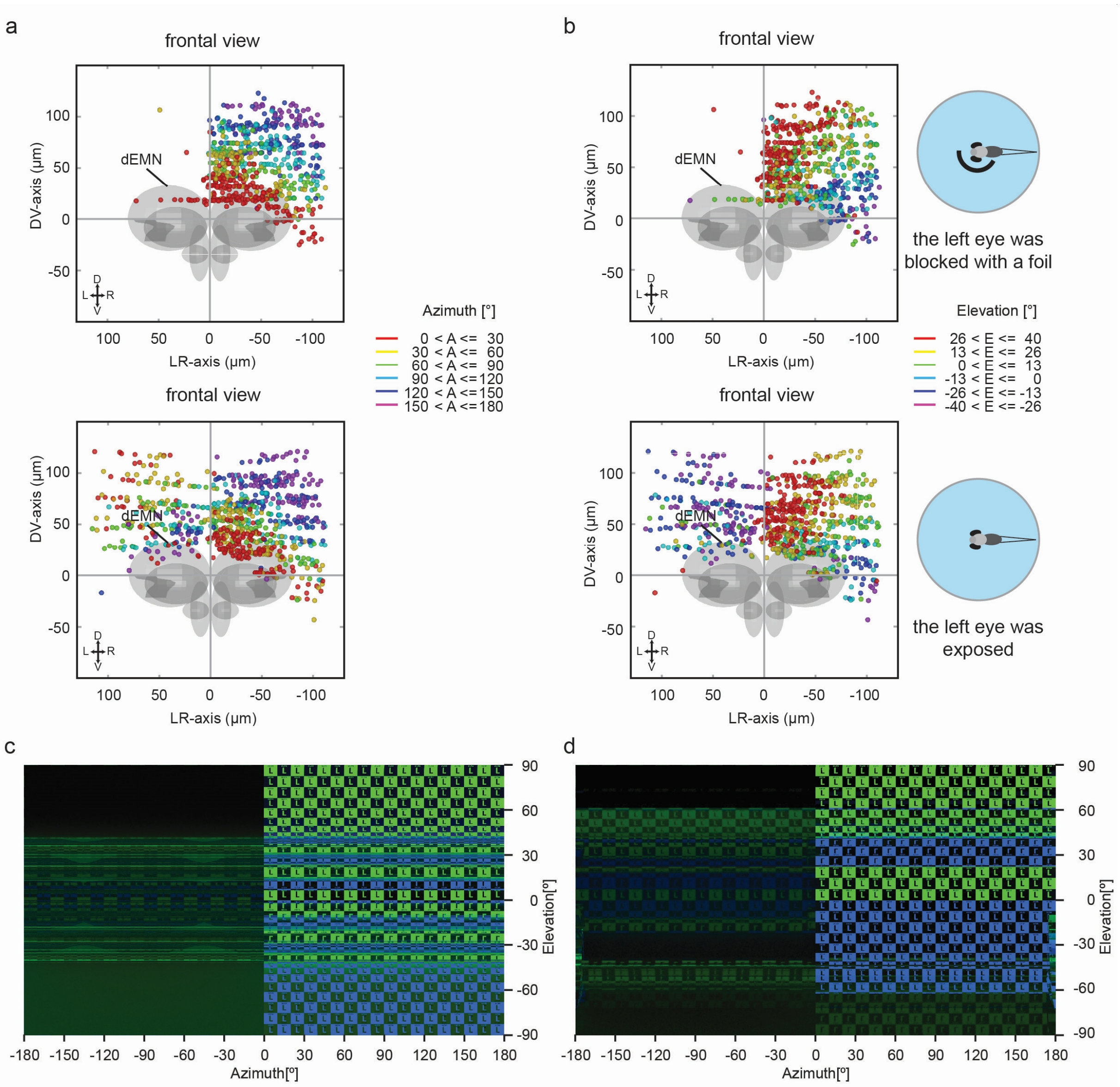

(**a, b**) Frontal views of the topographic maps of tectal small-size RF neurons in azimuth (a) or elevation (b). Each colored dot represents a single neuron with its receptive field center in the corresponding azimuth (a) or elevation (b) range. For example, all receptive field centers of the neurons in red are located between 0° azimuth (in front of the fish) and 30° azimuth on the nasal right side of the fish (a). For example, receptive field centers of the neurons in green are located slightly above the equator of the view filed (0° to 13° in elevation) (b). As indicated in the illustrations to the right (dorsal view), the left eye was covered by a black foil in the top row as the control group, while the left eye was exposed to potential stimulus artifacts in the bottom row (experimental animals). n = 6 fish, 3 composite brains. (**c, d**) Light reflection artifacts for the Petri dish lid (c) and the cylindrical container (d) using a monocular stimulus (cf. Figure 3b).

Figure S5. Other factors which influence the visual stimulus patterns perceived by the fish. Related to the discussion

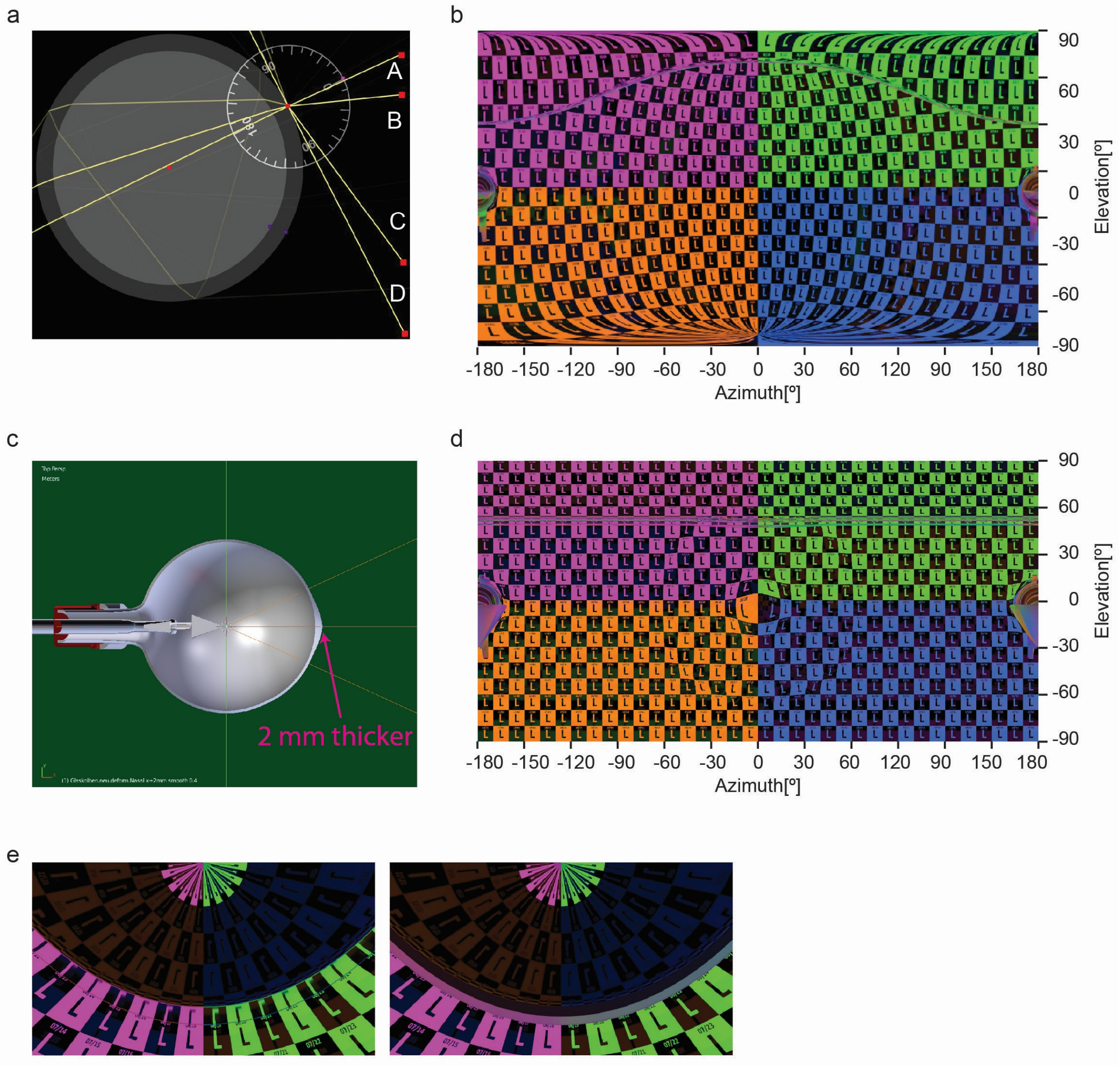

Figure S5. Other factors which influence the visual stimulus patterns perceived by the fish. Related to the discussion

(**a**) Four light beams (A, B, C and D) are shed into the glass bulb with different angles of incidence simulated with software (Ray Optics Simulation). The indices of refraction from water (inner circle) and glass (outer ring) are 1.333 and 1.47. The reflected light reaches the unstimulated eye (close to the center of the glass bulb) of the fish (left eye) only when the incidence angle of the visual stimulus is small (e.g., beam A). (**b**) An ideal checkerboard stimulus perceived when the animal is located far in front of the glass bulb center (15 mm = 38% of the radius). (**c**) Dorsal view of a simulated glass bulb with the frontal point 200% thicker (3 mm instead of 1 mm) than other regions of the glass wall. (**d**) An ideal checkerboard stimulus perceived by a fish when the animal is located in the center of the glass bulb shown in (c). (**e**) The material around the objective lens can cause additional stimulus reflections and it is advantageous to use objectives with non-reflective surfaces. Reflection of the stimulus pattern by microscope objective tips with different textures. Left side, glossy metal results in upside down reflection (upside down letters ‘L’); right side, Matt black and white, like Zeiss W N-Acroplan.

Figure S6. Experimental settings for the different containers 1

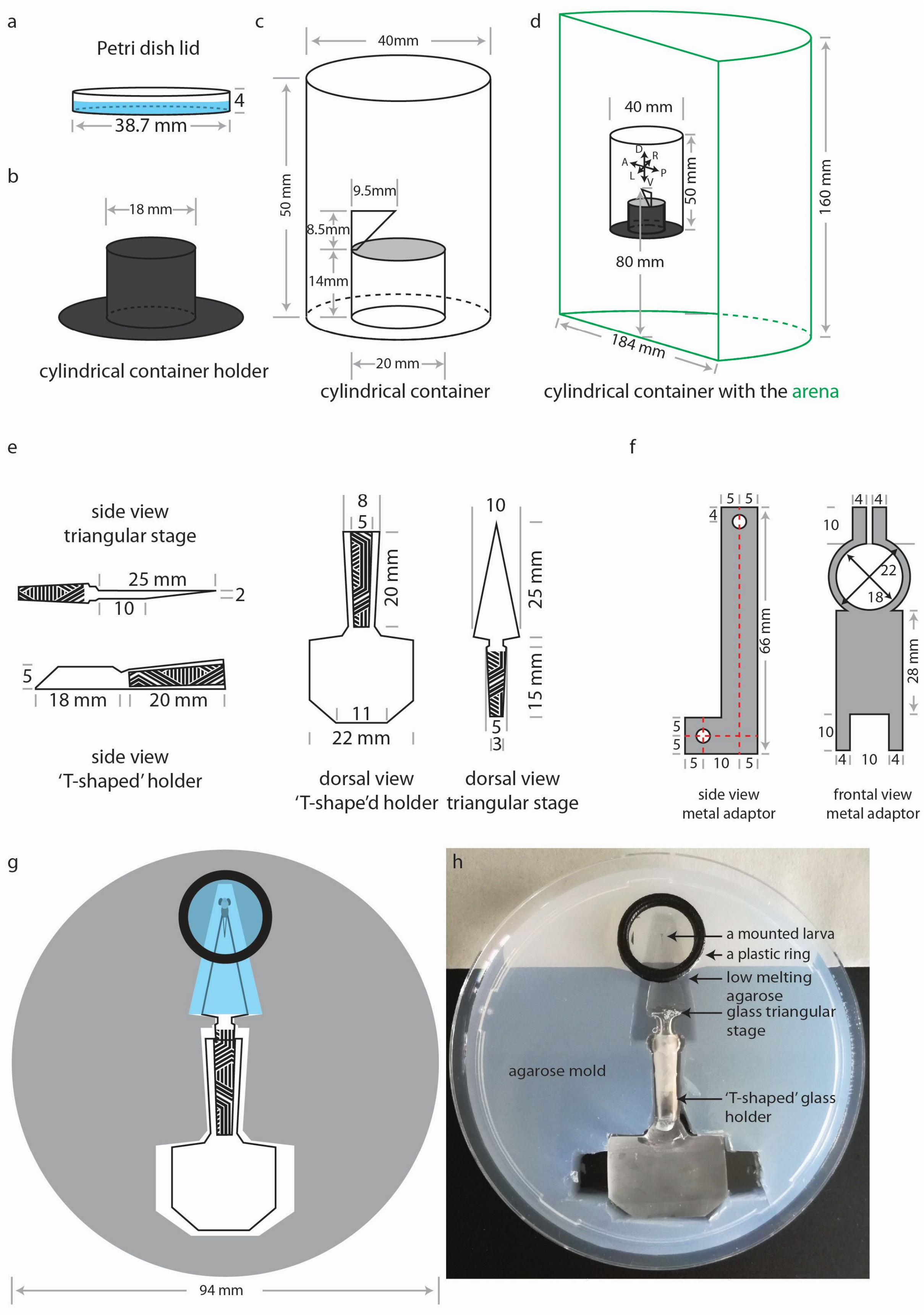

Figure S6. Experimental settings for the different containers 1

(**a**) A drawing of a Petri dish lid. (**b**) A drawing of the cylindrical container holder. (**c**) A drawing of the cylindrical container. (**d**) The drawing shows the locations of the cylindrical container and the LED half-cylindrical arena. (**e**) The side (on the left) and dorsal (on the right) view of the triangular stage and the 'T-shaped' holder. (**f**) The side (on the left) and frontal (on the right) views of the metal adaptor for fixing the glass bulb to the metal holder. (**g**, **h**) A larval zebrafish is embedded in low-melting agarose on the triangular stage before being transferred into the glass bulb during the experiment preparation.

Figure S7. Experimental settings for the different containers 2

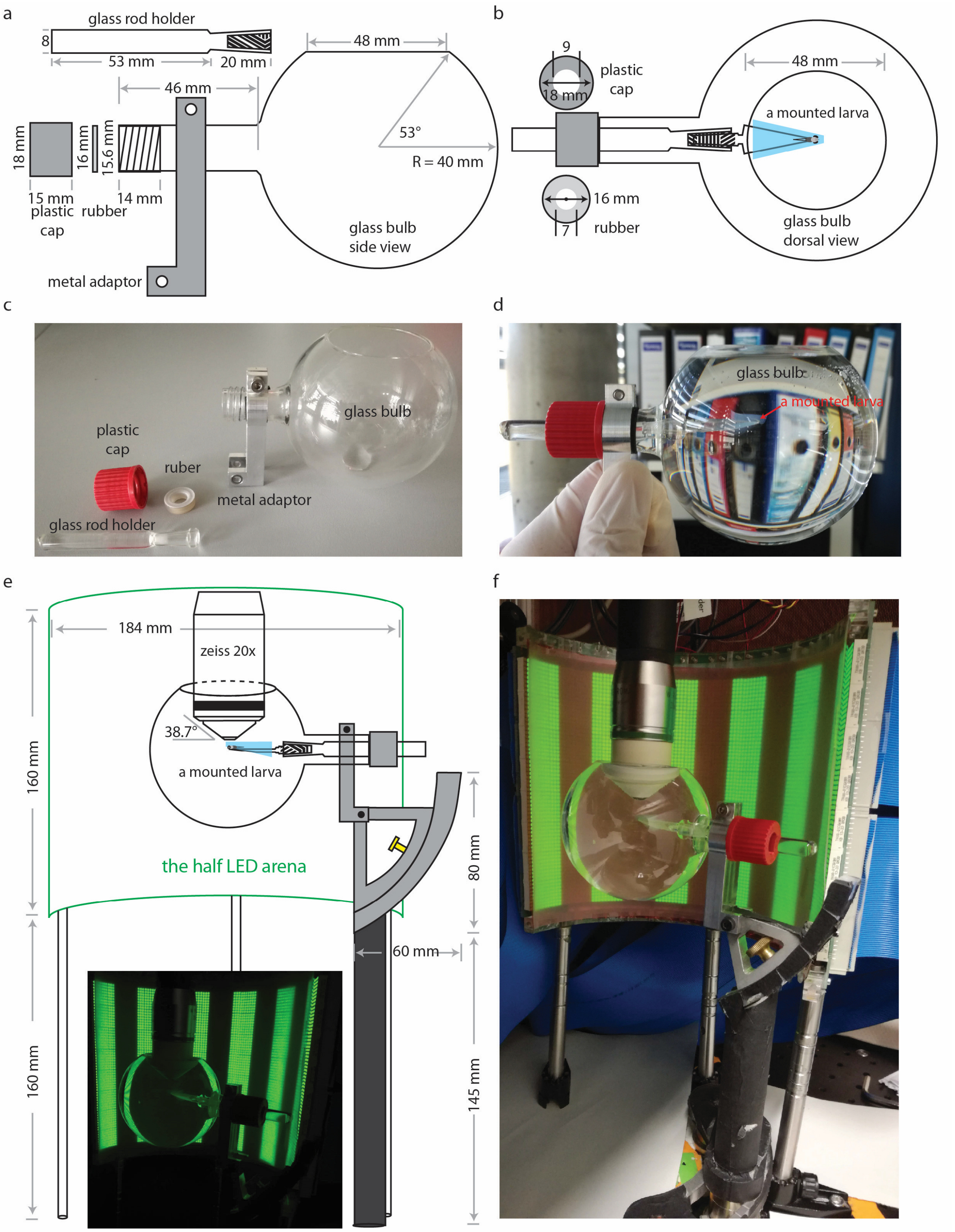

Figure S7. Experimental settings for the different containers 2

(**a**) Side view of the glass rod holder, plastic cap, rubber, metal adaptor and the glass bulb. (**b**) Dorsal view of the glass rod holder, plastic cap, rubber, and the glass bulb. (**c**) A photo of the glass rod holder, plastic cap, rubber, metal adaptor and the glass bulb. (**d**) A larval zebrafish is embedded in low-melting agarose on the triangular stage in the glass bulb. (**e**) A drawing shows the locations and sizes of the cylindrical container and the LED half cylindrical arena. (**f**) A photo shows the locations and sizes of the cylindrical container and the LED half cylindrical arena.
